# Clinical Features and Risk Factors for Atazanavir (ATV)-Associated Urolithiasis: A Case-Control Study

**DOI:** 10.1371/journal.pone.0112836

**Published:** 2014-11-19

**Authors:** Matthieu Lafaurie, Barbara De Sousa, Diane Ponscarme, Nathanael Lapidus, Michel Daudon, Laurence Weiss, Christophe Rioux, Erwan Fourn, Christine Katlama, Jean-Michel Molina

**Affiliations:** 1 Department of Infectious Diseases, Saint-Louis Hospital, Paris, France, and University of Paris Diderot Paris 7, Sorbonne Paris Cité, INSERM U941, Paris, France; 2 AP-HP, Hôpital Saint Louis, Service de Biostatistique et Information Médicale, Paris, France, and INSERM, UMR_S 1136, Institut Pierre-Louis d′Epidémiologie et de Santé Publique, Paris, France, and Sorbonne Universités, UPMC Univ Paris 06, UMR_S 1136, Institut Pierre-Louis d′Epidémiologie et de Santé Publique, Paris, France; 3 Laboratory of Clinical Physiology, Tenon Hospital, Paris, France; 4 AP-HP, Service of Clinical Immunology, Georges Pompidou European Hospital and University of Paris Descartes, Sorbonne Paris Cité, Paris, France; 5 Department of Infectious Diseases, Bichat Hospital, Paris, France; 6 Internal Medicine Department, Bicetre Hospital, Le Kremlin Bicetre, France; 7 Department of Infectious Diseases, Pitie-Salpetriere Hospital, Paris, France; Queen Mary Hospital, Hong Kong

## Abstract

**Objectives:**

Clinical features and risk factors for atazanavir (ATV)-associated urolithiasis have not been fully investigated.

**Methods:**

We reviewed all cases of ATV-containing urolithiasis identified by infrared spectrophotometry among HIV-infected patients over a 5-year period to describe their clinical features and outcome. A case-control study was performed to identify risk factors associated with ATV-associated urolithiasis using univariate and multivariate logistic regression analyses.

**Results:**

30 cases of ATV-associated urolithiasis were analyzed. Patients were mostly men (87%), median age: 45.5 years, median CD4 cell count: 443 cells/µL and 97% had plasma HIV RNA level <50 cp/mL. Median time between the initiation of ATV-containing regimen and the diagnosis of urolithiasis was 3.1 years. Patients presented with flank pain in 90% and macroscopic hematuria in 82.6%, 34% had renal dysfunction and 44.8% needed ureteroscopic treatment. In univariate analysis, chronic hepatitis C, a history of urolithiasis, prior use of indinavir, ATV duration, undetectable plasma HIV RNA, use of ritonavir as a booster and serum free bilirubin level were associated with ATV-urolithiasis. Multivariate models retained serum free bilirubin level (OR: 2.31, p<0.02) and either ATV duration (OR:  = 1.42, p = <0.03) or a history of urolithiasis (OR = 4.79, p<0.02) when adjusting on serum free bilirubin level as risk factors associated with urolithiasis.

**Conclusions:**

ATV-containing urolithiasis are associated with frank clinical symptoms and may require surgical intervention. A high serum bilirubin level, a long exposure to ATV and a history of urolithiasis are risk factors for this rare adverse event.

## Introduction

Atazanavir (ATV) is a widely used HIV protease inhibitor (PI) both in treatment-experienced and treatment-naive patients due to its potent antiviral activity and good safety profile [1]. Hyperbilirubinemia frequently occurs among patients receiving ATV due to the inhibition of the UGT1A1 metabolic pathway, but its clinical consequences are usually limited to mild scleral icterus. Other rare adverse events such as urolithiasis have been reported with ATV in postmarketing studies [2]. Indeed there have been a few case reports of urolithiasis in patients receiving ATV-based antiretroviral therapy (ART) [3]–[8]. Cohort studies have reported a high incidence rate of urolithiasis ranging from 7 to 24 cases per 1000 person-years among patients receiving ATV-based ART [9]–[10]. ATV/ritonavir (ATV/r) use was identified as an independent risk for renal stones (HR: 21.5, 95% confidence interval (CI): 2.9–160, p = 0.003) and may therefore represent a limitation for its use [10]. It is therefore critical to identify risk factors for ATV-associated urolithiasis in order to prevent or reduce the occurrence of this adverse event. Previous reports suggested several risk factors for ATV-induced renal stones, such as chronic renal impairment, co-infection with hepatitis virus, and past history of renal stones [7], [9]. However, the methods used in those studies were not adequate to determine risks factors for ATV/r-induced renal stones. Our aim was to better describe clinical features and outcome of patients with ATV-induced urolithiasis and to assess risk factors for this outcome using a case-control study.

## Methods

Patients with ATV-containing urolithiasis were identified from the computerized databases of three biochemistry laboratories, located in Paris from January 2008 to December 2012. In France HIV infected patients followed in hospitals are all registered in the French Hospital Database on HIV - FHDH and give written informed consent for their clinical records to be used in any epidemiological study.

In this study, patient records/information was anonymized and de-identified prior to analysis.

In all patients, the presence of ATV in the calculi was confirmed by infrared spectrometry [11]. Patients' charts were then reviewed after we obtained approval by the medical staff to participate in this study. For each case, three controls were sampled among patients who had no history of ATV-associated urolithiasis while treated with ATV-based regimens for at least 6 months (since this adverse event has been reported to occur late after treatment initiation). These controls were randomly selected from our computerized database of patients with HIV-infection at the Saint-Louis hospital in Paris. They were matched to their respective cases by choosing the closest medical consultation and biological sampling they had on the date of occurrence of ATV-associated urolithiasis in cases. The following data were obtained from medical charts: age, sexe, body mass index, conditions that predispose to urolithiasis (diabetes, obesity, drugs contributing to stone development such as sulfamide-containing coumpounds, serum calcium level), previous ART, current ART, duration of ATV exposure, CD4 T-cell count, plasma HIV-RNA level, CDC AIDS classification, prior history of urolithiasis before ATV initiation, clinical signs and symptoms of ATV-associated urolithiasis, imaging at diagnosis, composition of the stones, treatment and outcome. The following parameters were also collected in cases 6–12 months before the onset of urolithiasis, and 6–12 months later: serum creatinine, bilirubin, calcium, urine analysis.

Distributions in cases and controls were compared with the use of Mann-Whitney test for quantitative variables and Fisher's exact test for qualitative variables. Statistical tests were two-sided, and P values of less than 0.05 were considered to indicate statistical significance. Tests for paired samples were not used, as no other factor than the date of occurrence of ATV-associated urolithiasis was used to match cases and controls.

A risk factor analysis was conducted to identify factors associated with the occurrence of ATV-containing urolithiasis. We used logistic regression modeling in an univariate analysis among all covariates. Baseline characteristics available at ICU admission and associated with p values lower than 0.1 by univariate analysis or deemed clinically relevant were included in a multivariate logistic regression selection process. Given the number of cases, a maximum of three covariates was allowed in the tested models. Multiple imputation by chained equation was used to handle missing values. A stepwise Bayesian information criterion (BIC)-based selection was repeated in 30 imputed datasets to identify factors independently associated with urolithiasis. Models' calibrations were tested by the le Cessie–van Houwelingen goodness-of-fit test. Statistical analyses were performed with R version 3.0.2 (R Development Core Team 2013; R Foundation for Statistical Computing, Vienna, Austria).

## Results

Sixty-two ATV-containing urolithiasis cases were retrieved from the 3 biochemistry laboratories during the study period but only 30 cases could be analyzed. Patients not enrolled in our study were those who did not live in the Paris area rendering data collection uneasy. Characteristics of cases are depicted in Table 1. Most patients were male, with a median age of 45.5 years. Median duration of HIV infection was nearly 10 years. At diagnosis, median CD4 cell count was high and similar in both groups. Twenty-nine cases (96.7%) had plasma HIV RNA levels <50 cp/mL, versus 59/90 (65.6%) controls (p<0.001). Median time between the initiation of ATV-containing regimen and the diagnosis of urolithiasis was significantly longer in cases than in controls (3.1 years [range: 2.2–3.8] versus 1.9 [1.2, 3.2] respectively, p<0.009). In all but one case (96.7%) ATV daily dose was 300 mg boosted with ritonavir 100 mg whereas only 53 patients (58.9%) in the control group received ATV/r (p<0.001). The proportion of patients who received tenofovir, lamivudine or emtricitabine and abacavir was similar in both groups. There was no difference between cases and controls in terms of patients co-morbidities, use of drugs with lithogenic potential (data not shown) or serum calcium level. However, an history of prior use of indinavir, prior indinavir urolithiasis and also prior urolithiasis (any type) before ATV therapy were significantly more frequent in cases compared to controls (44.8% versus 20%, p<0.002; 26.7% versus 1.2%, p<0.001 and 36.7 versus 7.8%, p<0.01, respectively). Finally, median serum free bilirubin was significantly higher in cases compared to controls (49.1 mmol/l [IQR: 32.5, 56] and 30.9 [17.7, 45] respectively, <0.004).

**Table 1:** Baseline characteristics of HIV-infected patients with atazanavir-associated urolithiasis and controls.

	Controls (n = 90)	Cases (n = 30)	p
Male [n (%)]	68 (75.6%)	26 (86.7%)	0.31
Age (years) [median (IQR)]	47 [39, 52.8]	45.5 [42, 50.5]	0.85
Body Mass Index (BMI) (kg/m2) (median [IQR])	23 [21.1, 25.7]	22.8 [21.3, 24.2]	0.60
Diabetes [n (%)]	9 (10%)	1 (3.3%)	0.45
Duration of HIV infection (years) [median (IQR)]	12.0 [4.9, 18.3]	9.7 [4.2, 17.7]	0.47
CDC disease stage			0.20
A	48 (53.9%)	20 (66.7%)	
B	14 (15.7%)	1 (3.3%)	
C	27 (30.3%)	9 (30%)	
Nadir CD4 (cell count, cells/mL)	204 [112, 297]	209.5 [67, 313]	0.79
CD4+ cell count, cells/mL (IQR?)	492 [344, 657]	443 [401, 620]	0.77
Plasma HIV RNA >50 cp/mL [n (%)]	59 (65.6%)	1 (3.3%)	<0.001
Duration of ATV prior to urolithiasis (years) [median (IQR)]	1.9 [1.2, 3.2]	3.1 [2.2, 3.8]	<0.009
Associated ARV therapy [(n) %]			
Ritonavir (100 mg od) [(n) %]	68 (75.6%)	29 (96.7%)	<0.02
Lamivudine/emtricitabine	82 (91.1%)	26 (86.7%)	0.64
Abacavir	30 (33.3%)	10 (33.3%)	1.00
Zidovudine	6 (6.7%)	3 (10%)	0.69
Didanosine	11 (12.4%)	2 (6.7%)	0.51
Tenofovir	47 (52.8%)	16 (53.3%)	1.00
Baseline creatinine clearance* (median [IQR])	91.9 [77.5, 99.9]	81.5 [73.1, 98.2]	0.16
Chronic liver disease			
Hepatitis B	3 (3.3%)	1 (0.03%)	
Hepatitis C	2 (2.2%)	4 (13.3%)	
Any disease (including hepatitis)	8 (8.9%)	6 (20.0%)	
Chronic kidney disease** before urolithiasis [n (%)]	7 (7.9%)	4 (14.3%)	0.29
Prior treatment with indinavir (years) [(n) %]	18 (20%)	13 (44.8%)	<0.02
Duration of indinavir (months) (median [IQR])	0 [0, 0]	0 [0, 32]	<0.004
Previous history of indinavir-urolithiasis [n (%)]	1 (1.2%)	8 (26.7%)	<0.001
Previous history of urolithiasis before ATV therapy [n (%)]	7 (7.8%)	11 (36.7%)	<0.01
Serum calcium (mmol/L) (median [IQR])	2.3 [2.2, 2.4]	2.3 [2.3, 2.4]	0.96
Serum free bilirubin level (mmol/L) (median [IQR])	30.9 [17.7, 45]	49.1 [32.5, 56]	<0.004

Clinical presentation, treatment and outcome of patients with ATV-associated urolithiasis are described in Table 2. Infrared spectrometry showed that kidney stones contained a high proportion of ATV (median percentage of ATV: 89%, IQR: 59.0, 95.0). Most cases of ATV-urolithiasis were symptomatic with renal colic or lumbar/flank pain in 90% and gross hematuria in 82.6% of cases. Complications were also frequent, with bilateral involvement in 4 cases (14.3%), ureteral dilatation in 13 (54.2%) and renal failure (MDRD creatinine clearance <60 mL/min) in 9 (34.6%) patients. Intervention for stone removal was required in 13 patients (44.8%) and a double J ureteral stent was inserted in 8/28 cases (29%). ATV-associated urolithiasis led to ATV discontinuation in most patients (82.8%). Median creatinine clearance was significantly lower at diagnosis versus 6–12 months earlier (p<0.05) but returned to baseline, 6–12 months after urolithiasis onset (p = 0.26). However, in the 6 patients with renal failure at the time of ATV-urolithiasis, renal function did not normalize 6–12 months later. Urinary pH was measured in only 6 patients at the onset of urolithiasis and ranged from 5 to 6.9. Recurrence of urolithiasis during follow-up was reported in 3 patients, 2 of whom were still on ATV.

**Table 2:** Clinical presentation and outcome of 30 patients with ATV-associated urolithiasis.

Variable	n = 30
Signs and symptoms [n (%)]	
Renal colic/Lumbar and/or flank pain [n (%)]	27 (90)
Macroscopic hematuria [n (%)]	19 (82.6)
Créatinine clearance* [median (IQR)]	78.0 [45.6, 98.0]
Creatinine clearance* <60 mL/min [n (%)]	9 (34.6)
Stone size (mm) [median (IQR)]	5.5 [3.9, 9.3]
Stone composition percentage [median (IQR)]	
ATV	89.0 [59.0, 95.0]
Proteins	7.5 [3.0, 10.0]
Calcium oxalate	0 [0; 8]
Calcium phosphate	0 [0, 0]
Stone location [n (%)]	
Pyelic	6 (33.3)
Ureteral	11 (61.1)
Bladder	1 (5.6)
Ureteral dilatation [n (%)]	13 (54.2)
Bilateral urolithiasis [n (%)]	4 (14.3)
Treatment [n (%)]	
Medical	9 (31)
Lithotripsy	2 (6.9)
Urologic surgery	13 (44.8)
ATV discontinuation [n (%)]	24 (82.8)
Recurrence of urolithiasis [n (%)]	3 (10.7)
Créatinine clearance* 6–12 months after ATV-urolithiasis (mL/min) [median (IQR)]	84.5 [69.4, 96.8]

In univariate analysis, risk factors for ATV-urolithiasis were chronic hepatitis C, a history of urolithiasis, the prior use of indinavir, ATV duration, undetectable HIV plasma RNA, the use of ritonavir as a booster and the serum free bilirubin level (Table 3). Multivariate models retained serum free bilirubin level and either ATV duration or a history of urolithiasis as factors associated with ATV-associated urolithiasis (Table 4).

**Table 3:** Risk factors for atazanavir-associated urolithiasis, univariate analysis.

	OR	95% CI	p
Male sex	2.1	0.66–6.69	0.21
BMI (kg/m2)	0.93	0.84–1.04	0.24
Age (years)	1	0.95–1.04	0.83
Plasma HIV RNA <50 cp/mL	0.07	0–0.44	<0.01
RNA <50 cp/mL			
Previous history of urolithiasis	6.86	2.35–20.03	<0.001
Prior treatment with indinavir	3.25	1.33–7.96	<0.01
Duration of atazanavir (per year)	1.37	1.04–1.79	<0.03
Ritonavir (100 mg od)	9.38	1.21–72.9	<0.04
Associated antiretroviral therapy	0.8	0.34–1.85	0.60
Creatinine clearance (baseline)	0.99	0.97–1.01	0.39
Serum free bilirubin level (per 2-fold increase)	2.08	1.19–3.62	<0.01
Any chronic liver disease	2.56	0.81–8.11	0.11
Chronic hepatitis B	1.00	0.10–9.99	1
Chronic hepatitis C	6.77	1.17–39.07	<0.04

**Table 4:** Risk factors for atazanavir-associated urolithiasis, multivariate analysis.

	OR		95% CI	p
**MAIN MODEL WITH 3 VARIABLES**				
Duration of ATV (per year)		1.32	0.95–1.84	0.10
Serum free bilirubin level (per 2-fold increase)		2.31	1.18–4.52	<0.02
Previous history of urolithiasis		3.66	0.88–15.2	0.07
**ALTERNATIVE MODELS WITH 2 VARIABLES**				
Serum free bilirubin level (per 2-fold increase)		1.94	1.12–3.36	<0.02
Previous history of urolithiasis		4.79	1.44–15.98	<0.02
Duration of ATV (per year)		1.42	1.04–1.93	<0.03
Serum free bilirubin level (per 2-fold increase)		2.66	1.35–5.21	<0.01

## Discussion

We report here a large series of 30 cases of ATV-containing urolithiasis that allowed us to better described the clinical features and outcomes of this rare adverse event in HIV-infected patients receiving an ATV-based antiretroviral regimen.

Due to our strict definition of cases based on the identification of ATV in the calculi using infrared spectrometry we could ascertain that the stones obtained in our patients were indeed related to ATV use.

Patients with ATV-containing urolithiasis in our study were mostly men, with a median age of 45.5 years, a body mass index of 22.8 kg/m^2^, and a low prevalence of diabetes (3.3%). They were receiving an ATV-based regimen for a median of 3.1 years, a long duration of exposure which is consistent with previous reports [7]–[9]. The large majority of patients in our study were symptomatic with flank pain and macroscopic hematuria. However, the retrospective design of the study and our case-definition may have probably overlooked minor clinical forms since severe cases were more likely to be reported. Despite this limitation, it is striking to see that 34.6% of patients presented with renal failure (estimated creatinine clearance <60 ml/mn) and that more than half needed urologic intervention (lithotripsy or double J stent). Most patients discontinued ATV following the occurrence of this adverse event, and in those who continued treatment, the rate of relapse was high although the number of cases remains limited in our study. It seems reasonable though to discontinue ATV following an episode of ATV-containing lithiasis, due to the potential severity of the episode, the risk of relapse and the number of alternatives available. It seems indeed that this risk of urolithiasisurolithiasis is a distinct feature of some (atazanavir, indinavir) but not all PIs (lopinavir, darunavir) [9]–[10].

In a previous report, we showed that whereas lopinavir is not excreted in urine, pretty high concentration of ATV and darunavir can be achieved in urine, with the detection of ATV and darunavir crystals in a minority of patients [11]. However, the incidence of darunavir-associated urolithiasis is much lower than with ATV, and to our knowledge no darunavir-containing lithiasis has yet be reported [9]–[10]. The reasons for these differences between ATV and darunavir are yet unknown but could be related to differences in solubility of the PI-crystals, the role of urine pH (strong acidity is required to achieve optimal dissolution of ATV), and other factors.

Since ATV is a widely used PI, it is important to identify risk factors for urolithiasis. Our case-control study was able to identify the level of serum free bilirubin as an independent factor associated with a 2.31-fold increased risk of urolithiasis per 2-fold increase in serum level (Table 4). This finding is not unexpected as serum free bilirubin level is correlated to ATV plasma level, since ATV inhibits the UGT1A1 pathway [13]. Hyperbilirubinemia is indeed a frequent adverse event in patients receiving an ATV-based regimen, although the clinical consequences of hyperbilirubinemia except for jaundice are limited [1]. In a recent study, hyperbilirubinemia in patients receiving an ATV-based regimen was significantly associated with UGT1A1 rs887829 T allele, higher baseline bilirubin levels and slower plasma ATV clearance [14]. Also, ritonavir boost by inhibiting ATV clearance increases ATV exposure and bilirubin levels, explaining why ritonavir was found as a risk factor in the univariate analysis, and why 29 out of 30 cases were receiving a ritonavir-boosted ATV-based regimen. It is possible that unboosted ATV could be associated with a lower incidence of urolithiasis although more studies would be needed to confirm this hypothesis [15]. We were unfortunately unable to provide ATV plasma levels in our study to support this assumption.

In our study, a history of urolithiasis was also associated with an increased risk of ATV-containing urolithiasis. This finding can be explained by predisposing factors for the development of urolithiasis. Indeed, a number of patients already experienced indinavir-associated urolithiasis in our study, another PI that is prone to induce renal calculi and is also an inhibitor of the UGT1A1 pathway [16]. It seems therefore cautious to avoid the use of ATV among patients who have already experienced urolithiasis, and in particular among those who developed nephrolithiaisis while receiving indinavir.

Finally, the duration of ATV use was also associated with an increased risk of ATV-associated urolithiasis in our study. This finding could be explained by the time needed to form ATV-crystals and stones, and is consistent with previous reports underlining that ATV-associated urolithiasis were only reported after a couple of years of ATV-based therapy. This finding is however a matter of concern since it would imply that patients under ATV-based regimen with high serum free bilirubin levels carry a potential risk of developing urolithiasis over time.

In summary, our study has shown that ATV-containing urolithiasis can be associated with severe clinical symptoms and are associated with high and prolonged ATV. In those patients, most of them receiving a ritonavir-boosted ATV-based regimen, especially if they have a history of urolithiasis, a switch to another antiretroviral regimen should be considered to avoid the risk of urolithiasis.
